# Association between hyperuricemia and acute kidney injury in critically ill patients with sepsis

**DOI:** 10.1186/s12882-023-03129-x

**Published:** 2023-05-05

**Authors:** Yuan-Xia Jiang, Chun-Lei Gong, Yan Tang, Yang Yi, Fu-Gang Liu, Jing-Wen Zhou, Ying-Long Shi, Hong-Wei Zhou, Kai-Qing Xie

**Affiliations:** 1 Department of Blood Purification, The Second Affiliated Hospital of Guangxi Medical University, Nanning, 530007 China; 2 Department of Blood Purification, The First Affiliated Hospital of Guangxi Medical University, Nanning, 530021 China

**Keywords:** Hyperuricemia, Sepsis, Acute renal insufficiency, Risk-factors, Acute kidney injury

## Abstract

**Background:**

Sepsis-related AKI is related to short-term mortality and poor long-term prognoses, such as chronic renal insufficiency, late development of end-stage renal disease, and long-term mortality. In this study, we aimed to investigate the association of hyperuricemia with acute kidney injury (AKI) in patients with sepsis.

**Methods:**

The retrospective cohort study included 634 adult sepsis patients hospitalized in the intensive care unit (ICU) of the First Affiliated Hospital of Guangxi Medical University from March 2014 to June 2020 and the ICU of the Second Affiliated Hospital of Guangxi Medical University from January 2017 to June 2020. Based on the first serum uric acid level within 24 h of admission to the ICU, patients were divided into groups with or without hyperuricemia, and the incidence of AKI within seven days of ICU admission was compared between the two groups. The univariate analysis analyzed the effect of hyperuricemia on sepsis-related AKI, and the multivariable logistic regression model analysis was used.

**Results:**

Among the 634 patients with sepsis, 163 (25.7%) developed hyperuricemia, and 324 (51.5%) developed AKI. The incidence of AKI in the groups with and without hyperuricemia was 76.7% and 42.3%, respectively, with statistically significant differences (2 = 57.469, P < 0.001). After adjusting for genders, comorbidities (coronary artery disease), organ failure assessment (SOFA) score on the day of admission, basal renal function, serum lactate, calcitonin, and mean arterial pressure, hyperuricemia was showed to be an independent risk factor for AKI in patients with sepsis (*OR* = 4.415, 95%*CI* 2.793 ~ 6.980, *P* < 0.001). For every 1 mg/dL increase in serum uric acid in patients with sepsis, the risk of AKI increased by 31.7% ( *OR* = 1.317, 95%*CI* 1.223 ~ 1.418, *P* < 0.001).

**Conclusion:**

AKI is a common complication in septic patients hospitalized in the ICU, and hyperuricemia is an independent risk factor for AKI in septic patients.

## Introduction

Sepsis, a systemic inflammatory response syndrome characterized by physiological, pathological, and biochemical abnormalities caused by infection, is a typical critical illness in the ICU [1]. The incidence of sepsis is high, with over 19 million cases of sepsis worldwide each year, 750,000 new cases each year in the United States, and approximately 250,000 deaths from sepsis each year [2–4]. In China, it is estimated that over 5.6 million people develop sepsis each year, and over 1 million people die from it [5, 6]. Acute kidney injury (AKI) is one of the most common complications of sepsis, with an incidence of 40–50% in patients with sepsis [7–9]. Among the patients with sepsis, the mortality rate of patients with AKI is 50% higher than patients without AKI [8]. Sepsis-related AKI is associated with short-term mortality and poor long-term prognoses such as chronic renal insufficiency, late development of end-stage renal disease, and long-term mortality [10, 11].

The known risk factors for sepsis-related AKI are male, advanced age, decreased basal renal function, sequential organ failure assessment (SOFA), hyperlactatemia, hypoalbuminemia, hypotension, comorbidity with other underlying conditions such as diabetes mellitus, chronic kidney disease (CKD), cardiovascular disease, and liver disease [12–17]. However, the mechanisms of sepsis-related AKI are complex, and many risk factors remain unclarified. Uric acid is the end product of purine degradation and is excreted through the kidneys. Numerous previous epidemiological studies have shown that hyperuricemia is associated with the progression of hypertension, cardiovascular disease, diabetes mellitus, and CKD [17–20]. In addition, several studies have shown that uric acid can cause renal damage through various crystal-dependent and non-crystal-dependent mechanisms (secondary to vasoconstriction, oxidative stress, and inflammation) [20–23]. Hyperuricemia has been reported as an independent risk factor for CKD and AKI initiation and progression [24, 25].

The relationship between hyperuricemia and the risk of sepsis-related AKI is still unclear. Therefore, it is essential to investigate the association between hyperuricemia and the risk of sepsis-related AKI. In this study, we collected and analyzed clinical data comparing the incidence of AKI within seven days of ICU admission in patients with and without hyperuricemia. Then we examined the effect of Hyperuricemia on AKI initiation in patients with sepsis. This study aimed to investigate the effect of hyperuricemia on sepsis-related AKI and provide more references for the clinical management of sepsis-related AKI.

## Object and methodology

### Enrollment criteria

The clinical data of patients with sepsis admitted to the ICU of the Department of Internal Medicine of the First Affiliated Hospital of Guangxi Medical University from March 2014 to June 2020 and the ICU of the Second Affiliated Hospital of Guangxi Medical University from January 2017 to June 2020 were collected for a retrospective cohort study. Patients aged < 18 years, those with end-stage renal disease or renal transplantation, those with AKI or undergone renal replacement therapy (RTT) on the day of admission to the ICU, those whose serum creatinine was not rechecked after admission to the ICU, those treated with aminoglycoside antibiotics and vancomycin, and those with incomplete information were excluded. A total of 634 patients with sepsis were included in this study. This study was approved by the Medical Ethics Committee of Guangxi Medical University [Approval Document No. Lun Audit 2020-KY (0102)].

### Diagnosis of sepsis

The diagnostic criteria for sepsis refer to the Sepsis 3 diagnostic criteria proposed by the European Society of Intensive Care Medicine (ESICM) and the American Society of Critical Care Medicine in 2016: infection or suspected infection plus an increase in SOFA score ≥ 2 points [26]. In this study, the SOFA score was calculated using the relevant indicators when the patient was admitted to the ICU.

### AKI diagnosis and staging criteria

AKI diagnosis and staging criteria were based on the 2012 Kidney Disease: KDIGO-SCr criteria [27]. In this study, AKI occurrence in sepsis patients was defined as AKI occurring within 7 d of ICU admission [15]. The baseline creatinine is defined as the creatinine within 7 d before entering ICU. If the patient did not detect the creatinine before entering ICU, the first serum creatinine within 24 h of ICU admission would be used as the baseline creatinine.

### Observation indicators and related definitions

(1) Clinical characteristics: gender, age, alcohol intake and smoking history, comorbidities, the day of ICU admission SOFA score, mean arterial pressure;

(2) Laboratory data: serum creatinine, uric acid, lactate, procalcitonin, C-reactive protein, white blood cell count, hemoglobin, and albumin for the first time within 24 h after admission to the ICU were used as baseline indicators. The baseline estimated glomerular filtration rate (eGFR) was calculated according to the Modification of Diet in Renal Disease study equation modified by the Chinese coefficient [28]. Baseline eGFR < 60 ml·min^-1^· (1.73m^2^)^−1^ was diagnosed as basal chronic kidney disease [29].

### Definition of hyperuricemia

Serum uric acid > 7 mg/dL in men and > 6 mg/dL in women were diagnosed with hyperuricemia, according to the 2013 Chinese expert consensus [30]. Patients were divided into hyperuricemia and non-hyperuricemia groups according to the first serum uric acid value within 24 h after the ICU admission.

### Statistical methods

Statistical analysis was performed using SPSS 23.0 software. The measurement data were first tested for normality. Data conforming to a normal distribution are expressed as mean ± standard deviation (*x ¯* ± *SD)*, and their comparison between groups was performed using the two independent samples t-test. Data that were not normally distributed were expressed as median (quartiles) [*M (IQR, P25-P75)*], and their comparison between groups was performed using the Mann-Whitney *U* test. Counting data were defined as frequency (percentage or composition ratio), and measuring data were compared between groups. The Chi-square or Fisher’s exact probability test was used to compare groups. The Mann-Whitney U test was used to compare the grade data between the two groups. The risk factors for sepsis-related AKI were analyzed by univariate analysis. The multivariable logistic regression model analysis included indicators with *P* < 0.05 in the univariate analysis. *P* < 0.05 was considered a statistically significant difference.

## Results

### Basic characteristics of the study population

**Fig. 1:**
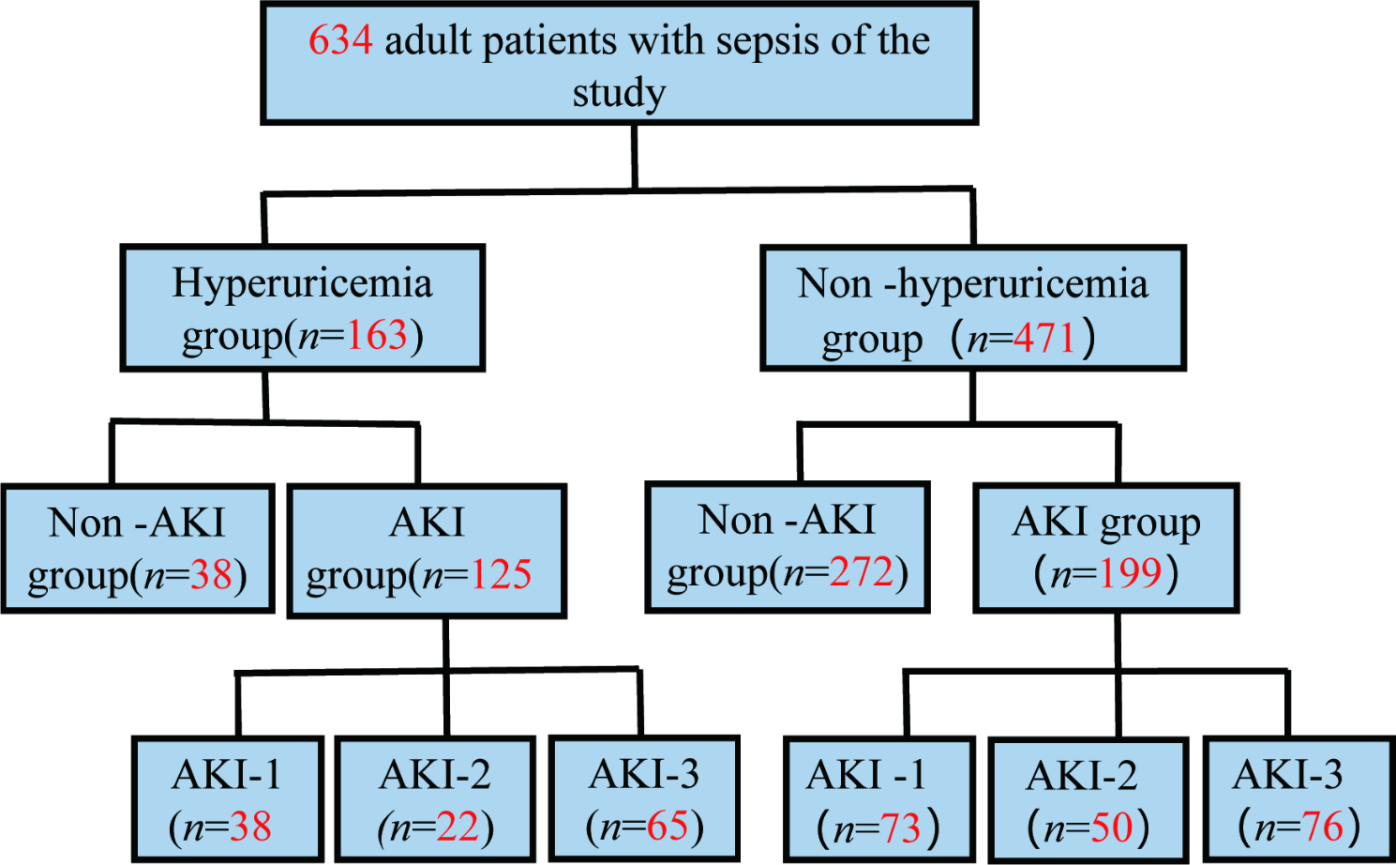
Schematic diagram of study grouping

Among the 634 patients with sepsis, the age was 64.0 (*IQR*, 50.0–75.0), the oldest being 99 years and the youngest 18 years. 411 (64.8%) males and 233 (35.2%) females. Two hundred thirteen patients (33.6%) had a smoking history, and 188 (29.7%) had a history of alcohol intake. The comorbidities were, in order of prevalence, 223 (35.2%) cases of hypertension, malignancy in 172 cases (27.1%), cerebrovascular disease in 169 cases (26.7%), diabetes mellitus in 144 cases (22.7%), chronic obstructive pulmonary disease in 65 cases (10.3%), coronary artery disease (CAD) in 58 cases (9.1%), and liver disease in 47cases (7.4%). The Baseline serum creatinine was 81.0 (*IQR*, 63.0–97.0) µmol/L, and baseline eGFR was 84.1 (*IQR*, 75.0–119.0) ml·min^− 1^· (1.73m^2^)^−1^, basal chronic kidney disease in 85 cases (13.4%). The baseline serum uric acid was 4.6 (*IQR*, 3.0-6.7) mg/dL; the SOFA score on ICU admission was 7.0 (*IQR*, 4.0–9.0). There were 163 patients (25.7%) with hyperuricemia. A total of 324 patients (51.1%) had AKI within seven days of admission to the ICU, and 111 (34.3%) had AKI stage 1, 71 (22.2%) had AKI stage 2, 141 (43.5%) had AKI stage 3. The grouping situation in this study is shown in Fig. 1.

### Comparison of clinical indicators between patients in the hyperuricemia group and the non-hyperuricemia group

Among the 634 patients, 163 were in the hyperuricemia group, and 471 were in the non-hyperuricemia group. The comparison of clinical indicators between the two groups is shown in Table 1. By chi-square test, two independent sample T-test, or rank-sum test for comparison of two independent samples, compared with patients in the non-hyperuricemia group, patients in the hyperuricemia group with hypertension, coronary artery disease, the proportion of basal chronic kidney disease, serum creatinine, lactate level, and white blood cell count were higher. Still, the baseline eGFR was lower in the hyperuricemia group, all with *P* < 0.05. AKI occurred in 125 patients in the hyperuricemia group (76.7%) and 199 patients (42.3%) in the non-hyperuricemia group, with statistically significant differences (*χ*^2^ = 57.469, *P* < 0.001). There were 38 (30.4%), 22 (17.6%), and 65 (52.0%) patients with AKI stages 1, 2, and 3 in the hyperuricemia group. And there were 73 (36.7%), 50 (25.1%), and 76 (38.2%) patients with stage 1, 2, and 3 AKI in the non-hyperuricemia group. The proportion of AKI-3 patients in the hyperuricemia group was higher with a statistical difference ( *Z* = -2.056, *P* = 0.040).

**Table 1:** Comparison of clinical indicators between the hyperuricemia group and non-hyperuricemia group

Variables	Hyperuricemia group (*n* = 163)	Non-hyperuricemia group (*n* = 471)	*χ*^2^/*z*/*t*	*P*
Age (years)^a^	65.0(*IQR*, 50.0–77.0)	63.0(*IQR*, 50.0–75.0)	-0.734	0.463
Male, *n* (%)	100(61.3)	311(66.0)	1.163	0.281
Alcohol intake history, *n* (%)	45(27.6)	143(30.4)	0.440	0.507
Smoking history, *n* (%)	53(32.5)	160(34.0)	0.115	0.735
With Hypertension, *n* (%)	70(42.9)	153(32.5)	5.811	0.016
With Diabetes, *n* (%)	41(25.2)	103(21.9)	0.744	0.388
With COPD, *n* (%)	11(6.7)	54(11.5)	2.928	0.087
With Cerebrovascular disease, *n* (%)	46(28.2)	123(26.1)	0.275	0.600
With Liver disease, *n* (%)	9(5.5)	38(8.1)	1.144	0.285
With Malignant tumor, *n* (%)	35(21.5)	137(29.1)	3.552	0.059
With CAD, *n* (%)	25(15.3)	33(7.0)	10.112	0.001
SOFA score(points) ^a^	7.0(*IQR*, 5.0–10.0)	7.0(*IQR*, 4.0–9.0)	-0.621	0.535
MAP (mmHg) ^b^	92.0 ± 22.4	90.4 ± 21.4	-0.795	0.427
Occurrence of AKI, *n* (%)	125(76.7)	199(42.3)	57.469	< 0.001
Basal chronic kidney disease, *n* (%)	44(27.0)	41(8.7)	34.889	< 0.001
Baseline serum creatinine (µmol/L) ^a^	88.0(*IQR*, 71.0-110.0)	78.0(*IQR*, 59.0–93.0)	-5.742	< 0.001
Baseline eGFR (ml/min/1.73m^2^) ^a^	78.5(*IQR*, 56.8–88.0)	87.8(*IQR*, 77.2-125.8)	-5.953	< 0.001
Lactate (mmol/L) ^a^	3.0(*IQR*, 1.7–4.9)	2.2(*IQR*, 1.3–3.9)	-3.595	0.003
Procalcitonin (ng/mL) ^a^	1.7(*IQR*, 0.4–11.2)	1.3(*IQR*, 0.4–7.3)	-1.416	0.157
C-reactive protein (mg/L) ^a^	73.2(*IQR*, 23.5-143.1)	90.1(*IQR*, 34.7-182.1)	-1.853	0.064
Leukocyte count (x10^9^/L) ^a^	14.0(*IQR*, 8.7–19.5)	11.6(*IQR*, 7.7–18.7)	-2.377	0.017
Hemoglobin (g/L) ^a^	102.0(*IQR*, 81.4-127.9)	97.9(*IQR*, 81.0-118.9)	-1.645	0.100
Serum albumin (g/L) ^a^	31.3(*IQR*, 26.4–35.5)	30.1(*IQR*, 25.9–34.4)	-1.519	0.129

COPD: Chronic obstructive pulmonary disease; CAD: coronary artery disease; SOFA: Sequential organ failure assessment; MAP: Mean arterial pressure; eGFR: estimated glomerular filtration rate. ^a^ is *M (IQR, P25-P75)*; ^b^ is *x* ®±*s*

### Analysis of risk factors for sepsis-related AKI

All patients were divided into AKI and non-AKI groups, and univariate analysis was performed by chi-square test or rank-sum test to compare two independent samples. The results suggested that compared with those in the non-AKI group, patients in the AKI group were more likely to be male, with coronary artery disease, basal chronic kidney disease, and SOFA score, baseline serum creatinine, uric acid, lactate, and procalcitonin levels were higher. Still, baseline eGFR and mean arterial pressure were lower, with statistically significant differences (all *P* < 0.05). There were 125 cases (38.6%) of hyperuricemia in the AKI group and 38 cases (12.3%) of hyperuricemia in the non-AKI group, with a statistically significant difference (*χ*^2^ = 57.469, *P* < 0.001). The baseline serum uric acid values were 5.7 (*IQR*, 4.1–8.7) mg/dL in the AKI group and 3.6 (*IQR*, 2.1-5.0) mg/dL in the non-AKI group, with a statistically significant difference (*Z* = -10.216, *P* < 0.001), as detailed above in Table 2.

**Table 2:** Univariate analysis of risk factors for sepsis-associated AKI

Variable(s)	AKI group (*n* = 324)	Non-AKI group (*n* = 310)	*χ*^2^/*z*	*P*
Age (years)^a^	64.5(*IQR*, 51.0-75.8)	63.0(*IQR*, 49.8–75.0)	-0.835	0.404
Male, *n* (%)	230(71.0)	181(58.4)	11.031	0.001
Alcohol intake history, *n* (%)	105(32.4)	83(26.8)	2.410	0.121
Smoking history, *n* (%)	110(34.0)	103(33.2)	0.037	0.847
With Hypertension, *n* (%)	124(38.3)	99(31.9)	2.789	0.095
With Diabetes, *n* (%)	75(23.1)	69(22.3)	0.071	0.789
With COPD, *n* (%)	29(9.0)	36(11.6)	1.220	0.269
With Cerebrovascular disease, *n* (%)	81(25.0)	88(28.4)	0.930	0.335
With Liver disease, *n* (%)	28(8.6)	19(6.1)	1.458	0.227
With Malignant tumor, *n* (%)	97(29.9)	75(24.2)	2.645	0.104
With CAD, *n* (%)	41(12.7)	17(5.5)	9.800	0.002
SOFA score(points) ^a^	8.0(*IQR*, 5.0–11.0)	6.0(*IQR*, 4.0–8.0)	-6.428	< 0.001
MAP (mmHg) ^a^	88.3(*IQR*, 72.3-104.7)	94.3(*IQR*,78.9-105.3)	-2.723	0.006
Hyperuricemia, *n* (%)	125(38.6)	38(12.3)	57.469	< 0.001
Basal chronic kidney disease, *n* (%)	62(19.1)	23(7.4)	18.733	< 0.001
Baseline serum creatinine (µmol/L) ^a^	88.0(*IQR*,71.0–97.0)	71.0(*IQR*, 56.0-89.3	-6.459	< 0.001
Baseline eGFR (ml/min/1.73m^2^) ^a^	81.1(*IQR*, 73.0-102.3)	91.5(*IQR*, 78.0-138.5)	-5.714	< 0.001
Uric acid (mg/dL) ^a^	5.7(*IQR*, 4.1–8.7)	3.6(*IQR*, 2.1-5.0)	-10.216	< 0.001
Lactate (mmol/L) ^a^	3.1(*IQR*, 1.7–5.7)	1.9(*IQR*, 1.2–3.1)	-6.486	< 0.001
Procalcitonin (ng/mL) ^a^	3.5(*IQR*, 0.7–20.0)	0.7(*IQR*, 0.2–2.1)	-9.469	< 0.001
C-reactive protein (mg/L) ^a^	92.5(*IQR*, 36.7- 191.8)	81.6(*IQR*, 31.3-167.7)	-1.584	0.113
Leukocyte count (x10^9^/L) ^a^	13.2(*IQR*, 7.9–19.7)	11.7(*IQR*, 7.9–17.5)	-1.378	0.168
Hemoglobin (g/L) ^a^	95.7(*IQR*, 78.1-119.6)	102.7(*IQR*, 83.0-122.0)	-1.786	0.074
Serum albumin (g/L) ^a^	29.8(*IQR*, 25.2–34.2)	30.9(*IQR*, 26.3–34.8)	-1.527	0.127

COPD: Chronic obstructive pulmonary disease; CAD: coronary artery disease; SOFA: sequential organ failure assessment; MAP: Mean arterial pressure; eGFR: estimated glomerular filtration rate. ^a^*is M (IQR, P25-P75)*.

The indicators with *P* < 0.05 in the univariate analysis were included in the multivariable logistic regression model analysis. The results showed that hyperuricemia, basal chronic kidney disease, serum lactate level, male, with coronary artery disease, SOFA score, and procalcitonin levels were independent risk factors for AKI in sepsis patients (all *P* < 0.05). The risk of AKI in patients with sepsis was 4.415 times higher in the hyperuricemia group than in the non-hyperuricemia group (*OR* = 4.415, 95%*CI* 2.793 ~ 6.980, *P* < 0.001), as shown in Table 3. The inclusion of specific serum uric acid values in the multivariable logistic regression model analysis showed that for every 1 mg/dL increase in serum uric acid values, the risk of AKI in sepsis patients increased by 31.7% (*OR* = 1.317, 95%*CI* 1.223 ~ 1.418, *P* < 0.001).

**Table 3:** Multivariable logistic regression model analysis of risk factors for sepsis-associated AKI

Variable(s)	*β*	*S.E.*	*OR*	95%*CI*	*P*
Hyperuricemia	1.485	0.234	4.415	2.793∼6.980	< 0.001
MAP(mmHg)	0.002	0.005	1.002	0.992∼1.011	0.734
Basal chronic kidney disease	0.742	0.303	2.099	1.159∼3.801	0.014
lactate (mmol/L)	0.063	0.030	1.065	1.004∼1.129	0.035
Male	0.863	0.202	2.371	1.597∼3.520	< 0.001
With CAD	0.945	0.347	2.572	1.303∼5.075	0.006
SOFA score (points)	0.149	0.031	1.160	1.091∼1.234	< 0.001
Procalcitonin (ng/mL)	0.036	0.008	1.037	1.012∼1.052	< 0.001

MAP: Mean arterial pressure; CAD: coronary artery disease; SOFA: Sequential organ failure assessment

## Discussion

Sepsis remains a significant burden in ICUs worldwide, with the incidence increasing every year, and AKI is a common complication of sepsis with an incidence of 40–50% [7–9]. In this study, the incidence of AKI in sepsis patients was 51.1%. AKI increases the length of hospital stay, medical costs, and risk of death in patients with sepsis and increases the risk of long-term CKD or even end-stage renal disease [8, 17, 31, 32]. Early identification and intervention of risk factors for sepsis-related AKI are essential to reduce AKI risk. In recent years, the number of risk factors for sepsis-related AKI has increased, and uric acid has received attention as a risk factor for the initiation and progression of CKD and AKI [24, 25]. However, there are still few studies on uric acid and sepsis-related AKI. Akbar et al. [14] conducted a prospective cohort study of 144 sepsis patients in the ICU, suggesting that Elevated serum uric acid levels were associated with poor sepsis prognoses and could increase their risk of AKI. However, this study was a single-center study with a small sample size, and these limitations may have influenced the final findings to some extent. In contrast, our results in this study suggest that AKI is a common complication in ICU patients with sepsis and that hyperuricemia is an independent risk factor for the development of AKI in patients with sepsis.

Among the 634 sepsis patients included in this study, AKI occurred in 324 patients (51.1%), including 125 patients (76.7%) in the hyperuricemia group and 199 patients (42.3%) in the non-hyperuricemia group, with statistically significant difference (*P* < 0.001). After correcting for gender (male), comorbidities (coronary artery disease), SOFA score on the day of ICU admission, basal chronic kidney disease, serum lactate, procalcitonin, and mean arterial pressure, the analysis showed that hyperuricemia remained an independent risk factor for AKI in patients with sepsis. The risk of AKI was increased by 3.415-fold compared to non-hyperuricemia sepsis patients. The risk of AKI in patients with sepsis increased by 31.7% for every 1 mg/dL increase in serum uric acid value. Multifactorial regression analysis showed that males, basal chronic kidney disease, serum lactate level, procalcitonin, coronary artery disease, and SOFA score on the day of ICU admission were also independent risk factors for AKI in patients with sepsis.

The mechanisms underlying sepsis-related AKI are complex and are currently thought to include renal microcirculatory disorders (redistribution of blood flow in the intrarenal microcirculation), inflammation and oxidative stress, and adaptive responses of renal tubular epithelial cells, and release of particulates [7, 8, 33, 34]. Uric acid is the end product of purine metabolism and exists in the body as soluble uric acid and urate crystals. Studies have shown that uric acid causes renal injury through pro-inflammatory, pro-oxidative stress, induction of mitochondrial dysfunction, activation of the renin-angiotensin system causing renal vasoconstriction, and renal vascular endothelial damage [21, 35]. Therefore, high uric acid levels can exacerbate oxidative stress and inflammatory responses in cells in sepsis and cause alterations in renal microcirculation, thereby increasing the risk of AKI. The present study results suggest that uric acid may be involved in the pathogenesis of sepsis-related AKI, which is also consistent with the study by Akbar et al. [14].

Previous studies have shown that elevated serum creatinine levels at CKD or baseline were an independent risk factor for sepsis-related AKI [13, 36]. Elevated CKD or baseline serum creatinine levels suggest reduced renal reserve and the presence of chronic inflammation and immunodeficiency in CKD, leading to increased susceptibility to kidney injury. In sepsis, patients with underlying renal insufficiency have a reduced ability of the kidneys to clear inflammatory mediators, with a subsequent increased risk of AKI. Serum lactate can assess the hemodynamic status of severe patients, with elevated lactate levels suggesting poor tissue oxygenation, increased anaerobic metabolism, and inadequate organ perfusion. Studies have shown that serum lactate levels and lactate clearance are risk factors affecting the prognosis of sepsis [37]. In the present study, serum lactate level was an independent risk factor for sepsis-related AKI. In addition, our findings suggest that diuretic use is also a risk factor for developing sepsis-related AKI. Coronary artery disease affects heart function, which may cause insufficient kidney infusion and cause AKI. Higher SOFA scores and procalcitonin levels indicate that patients’ infection is heavier, increasing the incidence of AKI.

Some studies have shown that male is an independent risk factor for sepsis-related AKI [12, 38, 39], but the exact mechanism is unknown. It may be related to the hormonal composition of the body. Cerceo et al. found that women had a lower risk of poor renal prognosis and death in patients with severe sepsis and septic shock [40]. They speculated that this might be related to women’s hormone levels, which was supported by the increased risk of poor renal prognosis and death after menopause. Our study found that males and basal chronic kidney disease were independent risk factors for sepsis-related AKI. Previous studies have also shown that a lower basal eGFR is an independent risk factor for AKI in patients with infective endocarditis [41].

Shortcomings of this study: This is a retrospective study with some missing clinical information, such as lack of height and weight due to the bedridden status of a significant proportion of patients admitted to the ICU, and urine volume criteria were not included in this study for the diagnosis of AKI, and the baseline creatinine measured in the hospital may also underestimate the actual AKI incidence. Therefore, further large-sample, multicenter prospective clinical studies are necessary to explore the effect of hyperuricemia on sepsis-related AKI.

## Conclusion

In conclusion, AKI is a common complication in ICU patients with sepsis, and hyperuricemia is an independent risk factor for AKI in septic patients. Hyperuricemia may be involved in the pathogenesis of sepsis-related AKI, and interventions for hyperuricemia may effectively prevent AKI in patients with sepsis.

## Data Availability

The datasets used and analyzed during the current study are available from the corresponding author upon reasonable request.
